# Ling-Gui-Zhu-Gan Decoction Protects H9c2 Cells against H_2_O_2_-Induced Oxidative Injury via Regulation of the Nrf2/Keap1/HO-1 Signaling Pathway

**DOI:** 10.1155/2020/8860603

**Published:** 2020-11-30

**Authors:** Xiang Wang, Tongjuan Tang, Mengting Zhai, Ruirui Ge, Liang Wang, Jinling Huang, Peng Zhou

**Affiliations:** ^1^Graduate School of Anhui University of Chinese Medicine, Hefei, Anhui 230012, China; ^2^Department of Integrated Traditional Chinese and Western Medicine, Anhui University of Chinese Medicine, Hefei, Anhui 230012, China; ^3^Research Institute of Integrated Traditional Chinese and Western Medicine, Anhui Academy of Chinese Medicine, Hefei, Anhui 230012, China; ^4^Anhui Province Key Laboratory of Chinese Medicinal Formula, Anhui Academy of Chinese Medicine, Hefei, Anhui 230012, China

## Abstract

**Objectives:**

Ling-Gui-Zhu-Gan decoction (LGZGD) is a potentially effective treatment for heart failure, and it showed significant anti-inflammatory potential in our previous studies. However, its ability to ameliorate heart failure through regulation of oxidative stress response is still unknown. This study was aimed to investigate the protective effect of LGZGD-containing serum on H_2_O_2_-induced oxidative injury in H9c2 cells and explore the underlying mechanism.

**Methods:**

Eighteen rats were randomly divided into two groups: the blank control group and LGZGD group. The LGZGD group rats were administrated with 8.4 g/kg/d LGZGD for seven consecutive days through gavage, while the blank control group rats were given an equal volume of saline. The serum was extracted from all the rats. To investigate the efficacy and the underlying mechanism of LGZGD, we categorized the H9c2 cells into groups: the control group, model group, normal serum control (NSC) group, LGZGD group, LGZGD + all-trans-retinoic acid (ATRA) group, and ATRA group. Malonedialdehyde (MDA) and superoxide dismutase (SOD) were used as markers for oxidative stress. Dichlorodihydrofluorescin diacetate (DCFH-DA) staining was used to measure the levels of reactive oxygen species (ROS). The apoptosis rate was detected using flow cytometry. The expression levels of pro-caspase-3, cleaved-caspase-3, Bcl-2, Bax, Keap1, Nrf2, and HO-1 were measured using western blotting. The mRNA levels of Keap1, Nrf2, and HO-1 were measured using RT-qPCR.

**Results:**

The LGZGD attenuated injury to H9c2 cells and reduced the apoptosis rate. It was also found to upregulate the SOD activity and suppress the formation of MDA and ROS. The expression levels of pro-caspase-3 and Bcl-2 were significantly increased, while those of cleaved-caspase-3 and Bax were decreased in the LGZGD group compared with the model group. As compared with the model group, the LGZGD group demonstrated decreased Keap1 protein expression and significantly increased Nrf2 nuclear expression and Nrf2-mediated transcriptional activity. ATRA was found to reverse the LGZGD-mediated antioxidative and antiapoptotic effect on injured H9c2 cells induced by H_2_O_2_.

**Conclusion:**

Our results demonstrated that LGZGD attenuated the H_2_O_2_-induced injury to H9c2 cells by inhibiting oxidative stress and apoptosis via the Nrf2/Keap1/HO-1 pathway. These observations suggest that LGZGD might prevent and treat heart failure through regulation of the oxidative stress response.

## 1. Introduction

Heart failure (HF), the end stage of various cardiovascular diseases, is a clinical syndrome typically characterized by structural or/and functional cardiac abnormalities [1]. As per various epidemiological studies, around 38 million people worldwide suffer from HF, and this disease's prevalence continues to rise [2]. Although the recommended “golden triangle” treatment regime is proven to be effective, patients often show poor compliance because of the associated adverse effects [3, 4]. The risk of survival of the patients who are given standardized treatment under the current guidelines is high. Therefore, developing novel therapies targeting pathways and selecting appropriate therapeutic interventions are significant for HF treatment.

Experimental data and clinical evidence indicate that oxidative stress is an important risk factor that is involved in pathogenesis and development of HF [5, 6]. During oxidative stress, the balance between the production of reactive oxygen species (ROS) and endogenous antioxidants defense system is disturbed, which can lead to physiological conditions such as myocardial cellular damage, cardiomyocyte apoptosis, myocardial hypertrophy, systolic dysfunction, and arrhythmia [7]. Reduction of oxidative stress has a protective effect on the myocardium and is a potential strategy for prevention and treatment of HF [8]. At present, the NF-E2-related factor 2-(Nrf2-) Kelch-like ECH-associated protein 1 (Keap1) pathway is a well-characterized pathway involved in cellular defense against various types of oxidative damage [9, 10]. Studies have shown that modulating the Nrf2/Keap1 signaling pathway significantly attenuates HF [11].

Clinical reports have confirmed that traditional Chinese medicine (TCM) formulas are therapeutically effective against HF [12]. Ling-Gui-Zhu-Gan decoction (LGZGD), a TCM formula composed of *Poria cocos*, *Ramulus Cinnamomi*, *Rhizoma Atractylodis*, and *Radix Glycyrrhizae*, is a potentially effective treatment for HF as per the 2016 Chinese guidelines for treatment of HF [13]. Clinical trials have also demonstrated its efficacy in treating HF [14, 15]. LGZGD contains natural compounds that can target various pathological pathways implicated in heart diseases [16]. Previous studies have revealed that LGZGD effectively regulates the structure and function of the failing heart by inhibiting the myocardial inflammation injury, alleviating the myocardial fibrosis, and improving the microstructural remodeling [17–20].

Considering the role of oxidative stress in HF and the potential of LGZGD in ameliorating HF through regulating oxidative stress response, we established an oxidative damage cell model by using H_2_O_2_ to stress H9c2 cells. Its underlying mechanism was further explored by studying the Nrf2/Keap1/HO-1 signaling pathway, which is involved in oxidative stress. Our observations provided insights into the molecular mechanism of LGZGD in the treatment of HF.

## 2. Materials and Methods

### 2.1. Drugs and Reagents

The components of LGZGD include *Poria cocos* (12 g), *Ramulus Cinnamomi* (9 g), *Rhizoma Atractylodis* (9 g), and *Radix Glycyrrhizae* (6 g) in the ratio of 4 : 3 : 3 : 2 (dry weight). The herbal sources and the preparation method of LGZGD were described in detail in our previous study [21]. ATRA, penicillin, and streptomycin were obtained from Sigma-Aldrich (St. Louis, MO, USA). H9c2 cells were obtained from the American Tissue Culture Collection (ATCC, Rockville, MD, USA). Fetal bovine serum (FBS) was purchased from Hyclone (Logan, UT, USA). The DMEM medium was purchased from GIBCO, Invitrogen Corporation (NY, USA). RIPA lysis buffer, Tris HCl (pH 6.8), and 1.5 mol/L Tris HCl (pH 8.8) were purchased from Beytime (Shanghai, China). The PVDF membrane filter (0.45 *μ*m) was purchased from Millipore (Schwalbach, Germany). ECL luminescent liquate was purchased from Thermo Fisher Scientific (Pittsburgh, PA, USA). RT-qPCR kit, Trizol, and the SYBR Premix Ex Tap kit were purchased from Takara (Japan). Assay kits of MDA and SOD were obtained from Jiancheng Biotech (Nanjing, Jiangsu, China). The ROS test kit was purchased from Beytime (Shanghai, China).

### 2.2. Preparation of LGZGD-Containing Serum from Rats

Sprague Dawley rats (*n* = 18, male, weight 200 ± 20 g) were provided by the experimental animal center of Anhui Medical University. The rats were fed with certified standard diet and water. All the procedures were approved by the center of scientific research of Anhui University of Chinese Medicine. The rats were randomly divided into two groups: the blank control group and LGZGD group. The LGZGD group was administrated with LGZGD (8.4 g/kg/d) for seven consecutive days [21, 22], and the blank control group was given an equal volume of saline by gavage. Abdominal aorta blood was harvested from rats one hour after administration of LGZGD or saline. The serum was separated from the blood and heated in a water bath set at 56°C for 30 minutes. The serum was then filtered through a microporous membrane and stored at −80°C for future use. All rats were anesthetized with sodium pentobarbital to minimize the suffering and subsequently sacrificed by cervical dislocation after blood collection.

### 2.3. Cell Culture and Drug Treatment

H9c2 cells were cultured in DMEM with 10% fetal bovine serum and penicillin and streptomycin mixture (100 U/mL each) in a 37°C incubator with 5% CO_2_. To induce an optimal oxidative damage, we exposed the H9c2 cells to 100 *μ*M H_2_O_2_ for 6 hours. The cell viability was in the range of 50% and 60% in the pretest. The H9c2 cells were categorized into the following groups: (1) control group; (2) model group; (3) NSC group: cells were treated with 20% normal rat serum; (4) LGZGD group: cells were treated with 20% LGZGD-containing rat serum [22]. After 12 hour *in vitro* culture, the cells of all the groups except the control were treated with 100 *μ*M H_2_O_2_ for 6 hours.

To investigate the role of the Nrf2 pathway in the LGZGD-mediated protective effect on H_2_O_2_-induced H9c2 injury, H9c2 cells were divided into six groups: (1) control group; (2) model group; (3) NSC group; (4) LGZGD group; (5) LGZGD + ATRA group: cells were treated with 20% LGZGD-containing serum and 5 *μ*mol/L ATRA [23] for 12 hours, followed by treatment with H_2_O_2_ for 6 hours; (6) ATRA group: cells were treated with 5 *μ*mol/L ATRA in DMEM containing 10% FBS followed by treatment with H_2_O_2_ for 6 hours. The first four groups were treated in the same way as described above.

### 2.4. Detection of MDA and SOD Levels

H9c2 cells were seeded in 6-well plates at a density of 1 × 10^6^ cells/well, incubated at 37°C with 5% CO_2_ and operated according to the kit instructions. MDA and SOD levels were calculated by measuring each well's absorbance at wavelengths 530 and 450 nm using a microplate reader.

### 2.5. Determination of Intracellular ROS Levels

Intracellular ROS levels were determined using the membrane-permeable fluorescent probe 27′-dichlorodihydrofluorescein diacetate (DCFH-DA). The cover glass (22 × 22 mm) was soaked with 75% alcohol for 2 hours and then placed in a 6-well plate after evaporating the alcohol. H9c2 cells were maintained in 6-well plates (1 × 10^6^ cells/well) at 37°C with 5% CO_2_ until 50% of the cells fused. After the corresponding processing was carried out as per the groups, the cells were stained with 10 *μ*M of DCFH-DA for 40 minutes and mixed every 3–5 min. Furthermore, the cells were washed three times with serum-free DMEM before being examined and photographed using a Zeiss confocal microscope.

### 2.6. Detection of Apoptosis by Flow Cytometry

The apoptosis rate of H9c2 cells was detected using flow cytometry. In brief, the cells were harvested after experimental treatments, washed twice with cold PBS, and labeled with 5 *μ*L FITC and 5 *μ*L PI in the dark for 15 minutes at room temperature. The percentage of apoptotic cells was measured by the Beckman DxFlex flow cytometer (Beckman, USA) and analyzed using FlowJo 7.6.

### 2.7. Reverse Transcription-Quantitative Polymerase Chain Reaction (RT-qPCR)

Each group was prepared as a cell suspension inoculated in a 6-well plate at 1 × 10^6^ cells/well. Total RNA was extracted from different groups of H9c2 cells following the manufacturer's instructions using 0.5 mL TRIzol reagent. cDNA was synthesized from 2 *μ*L RNA using the 4 *μ*L 5 × iScript reaction mix, 1 *μ*L iScript reverse transcriptase, and 13 *μ*L nuclease-free water under the following conditions: 25°C for 5 minutes, 42°C for 30 minutes, and 85°C for 5 minutes. The qPCR reaction was performed using the ABI 7500 real-time fluorescent quantitative PCR instrument (ABI, USA) under the following conditions: 95°C for 10 minutes followed by 40 cycles of 95°C for 15 seconds, 60°C for 1 minute, and 72°C for 40 seconds. Keap1, Nrf2, and HO-1 mRNA levels were analyzed using the comparative 2^−ΔΔCt^ method normalized to GAPDH. The specific primers used are listed in Table 1.

### 2.8. Western Blot Analysis

The cytoplasmic and nuclear protein fractions were extracted from H9c2 cells and quantified with the BCA method. The proteins were added to each well and separated on a 4% to 12% polyacrylamide gradient gel. The separated proteins were transferred onto PVDF membranes. The membranes were blocked in 5% nonfat dry milk for 1 hour and incubated overnight at 4°C with the primary antibodies (Table 2). After carefully washing thrice with Tris-buffered saline and TBST, the membranes were incubated with HRP-conjugated secondary antibodies (Table 2) for 1 hour at room temperature. The protein bands were detected and examined using a chemiluminescence image analyzer. The mean gray values of the bands were analyzed by Image *J* software.

### 2.9. Statistical Analysis

The results are presented as mean ± standard deviation (SD). The statistical analysis was performed using SPSS 23.0 and Graphpad 6.0 software. Differences among multiple groups were evaluated using one-way analyses of variance (ANOVA) and Tukey's test. A *P* < 0.05 was treated as statistically significant.

## 3. Results

### 3.1. Effect of LGZGD on the Expression Levels of ROS, MDA, and SOD in H_2_O_2_-Treated H9c2 Cells

**Figure 1:**
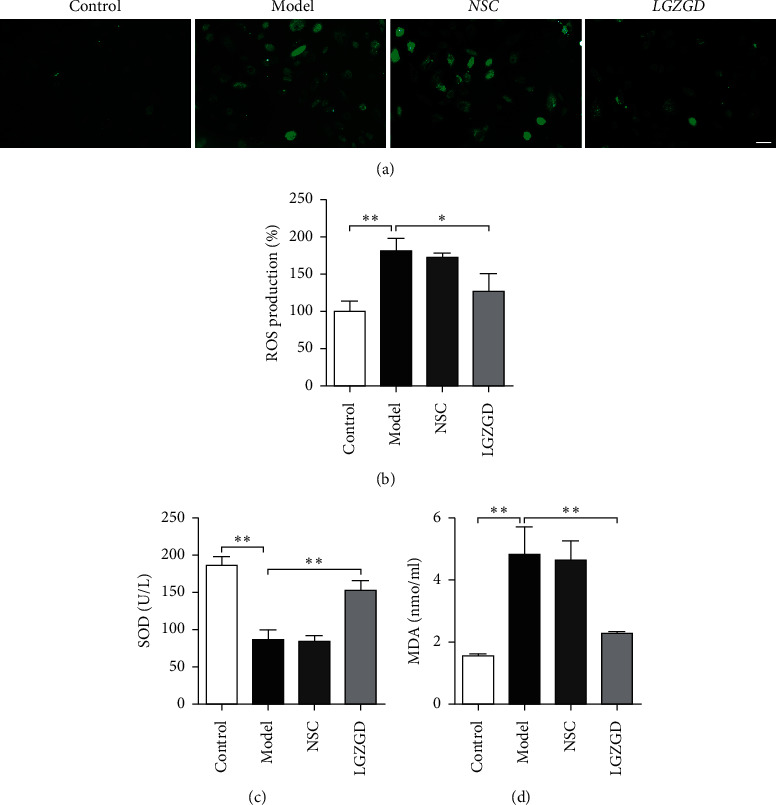
The antioxidative effect of LGZGD on H_2_O_2_-induced H9c2 cells. ROS production was detected by using the DCFH-DA probe using a Zeiss confocal microscope (a, b). The SOD activity (c) and MDA content (d) in H9c2 cells were detected using the SOD assay kit and MDA assay kit, respectively. All results are presented as mean ± SD for three independent experiments. *∗P* < 0.05, *∗∗P* < 0.01.

The expression levels of ROS and MDA significantly increased, while those of SOD sharply decreased in the model group compared with the control group (*P* < 0.01). Compared with the model group, the LGZGD group had remarkably reduced expression levels of ROS and MDA and significantly elevated levels of SOD (*P* < 0.01, *P* < 0.05) (Figure 1).

### 3.2. Effect of LGZGD on the Expression Levels of Apoptosis-Related Proteins and Apoptosis Rates in H_2_O_2_-Treated H9c2 Cells

**Figure 2:**
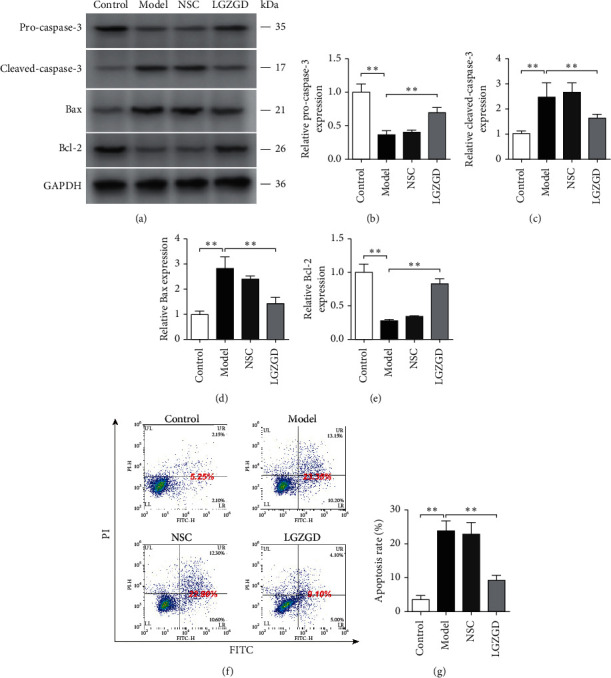
The antiapoptotic effect of LGZGD on H_2_O_2_-treated H9c2 cells. The expression levels of pro-caspase-3 (a, b), cleaved-caspase-3 (a, c), Bax (a, d), and Bcl-2 (a, e) in the indicated groups were detected by western blotting. Apoptosis rates in the indicated groups were detected using flow cytometry (f, g). All results are presented as mean ± SD for three independent experiments. *∗∗P* < 0.01.

In the model group, the expression levels of pro-caspase-3 and Bcl-2 were significantly lower, and those of cleaved-caspase-3 and Bax were significantly higher than the corresponding levels in the control group (*P* < 0.01) (Figure 2). On the contrary, treatment with LGZGD showed the opposite results. The apoptosis rate was significantly higher in the model group than in the control group (*P* < 0.01). In contrast, the apoptosis rate in the LGZGD group was significantly reduced (*P* < 0.01).

### 3.3. LGZGD Regulated the Expression Levels of Keap1, Nrf2, and HO-1 in H_2_O_2_-Treated H9c2 Cells

**Figure 3:**
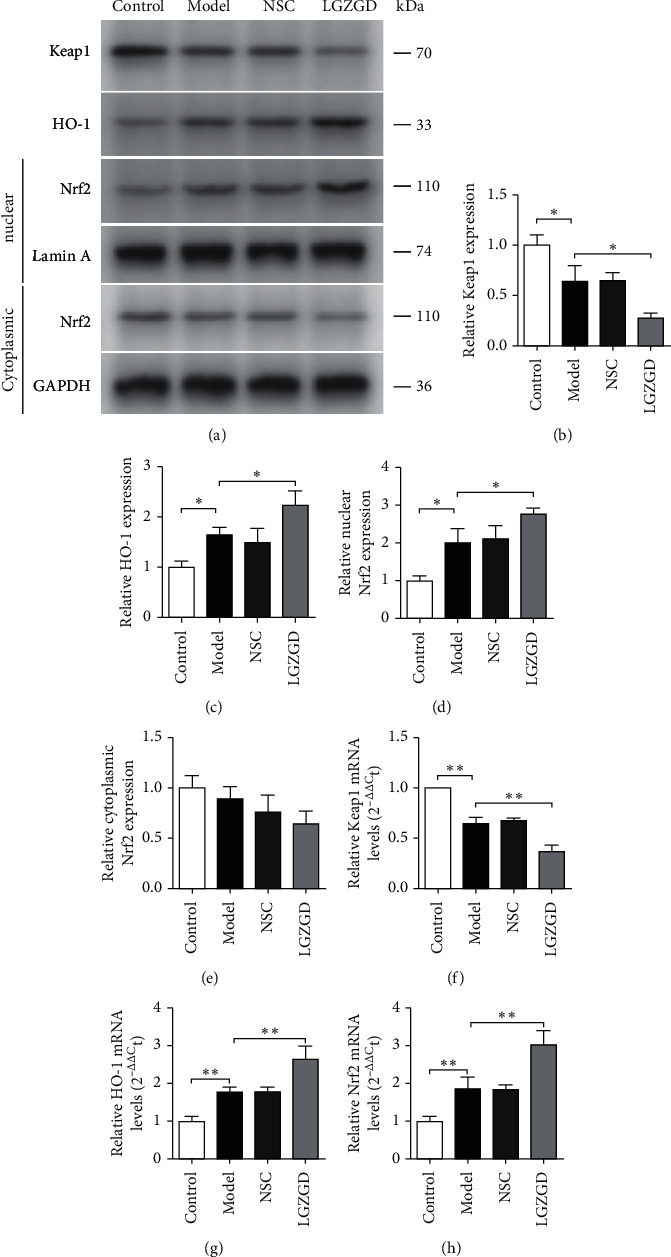
The expression of Keap1 (a, b), HO-1 (a, c), nuclear Nrf2 (a, d), and cytoplasmic Nrf2 (a, e) were detected by western blotting. The mRNA levels of Keap1 (f), HO-1(g), and Nrf2 (h) were detected by RT-qPCR. All results are presented as mean ± SD for three independent experiments. *∗P* < 0.05, *∗∗P* < 0.01.

The western blotting analysis showed that the protein expression levels of Keap1 were significantly lower in the model group than in the control group. Similarly, nuclear transport of Nrf2 and HO-1 was significantly higher in the model group than in the control group (Figure 3) (*P* < 0.05). As compared with the model group, the LGZGD treatment group had a lower expression of Keap1 and significantly increased nuclear transport of Nrf2 and HO-1 (*P* < 0.05). The mRNA expression levels of Keap1, HO-1, and Nrf2 were consistent with the protein expression levels (*P* < 0.01).

### 3.4. Nrf2 Pathway Is Associated with the Protective Effect of LGZGD-Mediated on H9c2 Cells Injury Induced by H_2_O_2_

**Figure 4:**
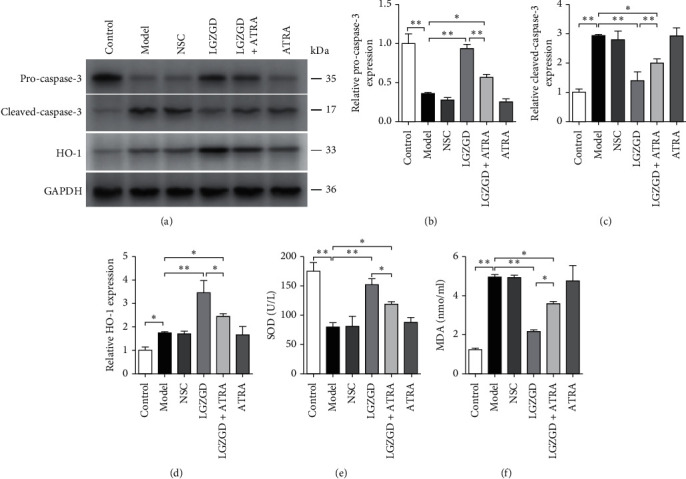
ATRA disrupts the protective effect of LGZGD in H_2_O_2_-treated H9c2 cells. The expression of pro-caspase-3 (a, b), cleaved-caspase-3 (a, c), and HO-1(a, d). The SOD activity (e) and MDA content (f) in H9c2 cells were detected by using the SOD assay kit and MDA assay kit, respectively. All results are presented as mean ± SD for three independent experiments. *∗P* < 0.05, *∗∗P* < 0.01.

To determine whether the protective effect of LGZGD was mediated by the Nrf2 pathway, cells were treated with an Nrf2 inhibitor (ATRA). As compared with the model group, the LGZGD group was found to have decreased MDA and cleaved-caspase-3 expression levels, increased SOD activity, and increased pro-caspase-3 and HO-1 expression levels (*P* < 0.01). Similarly, as compared with the LGZGD group, the LGZGD + ATRA group had increased MDA and cleaved-caspase-3 expression levels and decreased SOD activity and pro-caspase-3 and HO-1 expression levels (*P* < 0.01; *P* < 0.05) (Figure 4).

## 4. Discussion

### 4.1. Nrf2 Pathway and Cardiovascular Diseases

Oxidative stress contributes to cellular and molecular damage through excessive ROS generation [24, 25]. As a second messenger and signal transduction regulator, ROS can promote vascular endothelial cell proliferation, accelerate myocardial cell apoptosis and vascular inflammation, induce collagen production of myocardial fibroblasts, and cause irregular vascular remodeling, leading to the onset and progression of cardiovascular diseases [26, 27].

The Nrf2 defense system is highly sensitive to the changes in the cellular redox balance during the development of cardiovascular diseases [28]. This system can regulate endogenous antioxidant levels, detoxification enzymes, transcription factors, and growth factors to prevent cellular damage [29]. Among the signaling pathways that regulate Nrf2 and protect the cells against oxidative stress, the Nrf2/Keap1/HO-1 signal axis is the most critical. Nrf2 is a cytoprotective transcription factor. Once Nrf2 is activated, it effectively resists inflammation and apoptosis through the maintenance of cellular redox homeostasis, elimination of reactive oxidants, and regulation of apoptotic proteins [30, 31]. As per studies, mice with a knocked out Nrf2 gene have a left ventricular diastolic dysfunction and quickly transition from cardiac compensatory adaptation to HF [32, 33]. The overexpression of Nrf2 preserves the morphology and function of cardiomyocytes' mitochondria after they are treated with H_2_O_2_ [34]. Therefore, Nrf2 has the potential to protect against and attenuate cardiovascular diseases. The primary intracellular regulator of Nrf2, Keap1, plays a sensory role in detecting oxidative and electrophilic stresses [10]. Nrf2 and Keap1 are a part of the main regulatory system of cytoprotective enzyme genes. In physiological conditions, Nrf2 binds to Keap1 in the cytoplasm to maintain its stability. Under oxidative stress, Nrf2 is released from Keap1 and translocated to the nucleus, where it combines with ARE to activate the transcription of cellular defense genes (e.g., SOD, CAT, NQO1, and HO-1). HO-1 is a rate-limiting enzyme during the process of heme catabolism. It is expressed at low levels in the unstimulated state; however, when the cells are exposed to oxidants such as H_2_O_2_, heme, or hypoxic conditions, HO-1 can be activated. Enhanced expression of HO-1 maintains redox homeostasis against ROS generation and prevents cardiomyocyte injury from oxidative stress by converting mono-oxidant carbon (CO), ferrous (Fe^2+^), and biliverdin into bilirubin [35]. HO-1 overexpression has been proven to restore the coronary artery ligation-induced heart failure while HO-1 knockout accelerates the incidence and growth of atherosclerosis [36, 37]. Therefore, HO-1 plays a critical role in preserving cardiac homeostasis and attenuating heart injury. The modulation of Nrf2/Keap1/HO-1 signaling is known to regulate cardiac ischemia-reperfusion injury, hypertensive heart disease, coronary heart disease, and arrhythmia [38]. Modulation of Nrf2/Keap1/HO-1 signaling treats cardiovascular diseases by alleviating oxidative stress in cardiomyocytes, enhancing the cellular antioxidant defense system, and upregulating the viability of cardiomyocytes. We found that LGZGD protects myocardial tissues and prevents H9c2 cell injury by regulating the expression of NF-*κ*B; however, the upstream molecular factors, including Nrf2 and HO-1, have not been studied yet. Therefore, we utilized H_2_O_2_ to establish an oxidative stress injury model in H9c2 cardiomyocytes and investigated the protective effect of LGZGD and the underlying mechanism.

### 4.2. Active Components of the LGZGD and Nrf2/Keap1/HO-1 Pathway

The pharmacokinetics studies showed that LGZGD contains tumulosic acid, dehydrotumulosic acid, polyporenic acid C, cinnamic acid, atractylenolide I, atractylenolide II, atractylenolide III, glycyrrhizic acid, glycyrrhetinic acid, liquiritigenin, and isoliquiritin [39–41]. The main active components of LGZGD were found to have promising Nrf2 regulating activity. Cinnamic acid from *Ramulus Cinnamomi* promotes nuclear translocation of Nrf2 and induces HO-1 expression, thereby inhibiting UVA-induced ROS production [42]. Atractylenolide II from *Atractylodes macrocephala* Koidz significantly inhibits the ionizing radiation damage by enhancing the expression of HO-1 through the Nrf2 signaling pathway [43]. Glycyrrhetinic acid in *Radix Glycyrrhizae* decreases the expression of Bax, increases Bcl-2 expression, and upregulates Nrf2 and HO-1 levels to resist MTX-induced liver damage [44]. Glycyrrhizic acid from *Radix Glycyrrhizae* shows significant cardioprotective effects by upregulating HO-1 and Nrf2 expressions in ISO-induced myocardial ischemia [45]. Liquiritigenin from *Radix Glycyrrhizae* promotes Nrf2 nuclear translocation and increases the expression of HO-1, which was related to the Nrf2/Keap1/HO-1 signaling pathway [46].

### 4.3. LGZGD and Nrf2/Keap1/HO-1 Signaling Pathway

The intracellular ROS content, lipid peroxidation products such as MDA, and antioxidant enzymes such as SOD can indirectly affect ROS generation under oxidative stress. We investigated whether LGZGD possesses antioxidant activity. We selected H_2_O_2_ as an oxidative stress inducer since it stimulates the generation of free radicals and induces apoptosis. We clearly showed that pretreatment with LGZGD reduces MDA and ROS generation and improves the SOD activity. Our findings suggest that the protective effect of LGZGD on H_2_O_2_-induced H9c2 cell injury might be partly due to the improvement of oxidant and antioxidant balance. Apoptotic proteins (caspase 3, Bcl-2, and Bax) also play an essential role in the onset of cardiovascular diseases. In our study, LGZGD was found to significantly reduce the rate of apoptosis, decrease the expression levels of cleaved-caspase-3 and Bax, and increase the expression levels of pro-caspase-3 and Bcl-2. These observations highlight the antiapoptotic potential of LGZGD.

**Figure 5:**
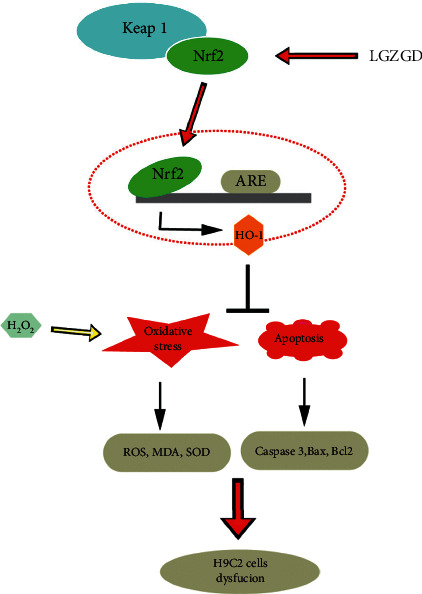
Mechanism of LGZGD acting on the Nrf2/Keap1/HO-1 signaling pathway.

Furthermore, LGZGD decreased the mRNA and protein expression of Keap1 and increased the Nrf2 translocation, nuclear Nrf2 expression, and HO-1 expression (Figure 5). To investigate the role of Nrf2 in protecting the cells from H_2_O_2_-induced injury, we used an Nrf2 inhibitor ATRA. ATRA was found to reverse the antioxidative and antiapoptotic effects of LGZGD. The results of this study confirmed our hypothesis that Nrf2 plays a crucial role in preventing H9c2 cell injury and indicated that LGZGD might protect H9c2 cells against injury induced by H_2_O_2_ via the regulation of the Nrf2/Keap1/HO-1 signaling pathway.

Our study has several limitations. The antioxidant mechanism of LGZGD was studied only *in vitro*, and no animal experiments were conducted to validate the results.

## 5. Conclusions

The present study is the first to report that the protective effects of LGZGD against H_2_O_2_-induced oxidative stress in H9c2 cells are mediated via the regulation of the Nrf2/Keap1/HO-1 pathway. In conclusion, the current data confirm that LGZGD exhibits remarkable protective effects on H_2_O_2_-induced injury in H9c2 cells by suppressing oxidative stress. Besides, LGZGD activates the antioxidant response of Nrf2 and inhibits apoptosis, which may be related to its protective effect. These findings indicate that LGZGD could act as a potentially effective drug for the treatment of HF. Furthermore, *in vivo* studies directed towards the Nrf2/Keap1/HO-1 pathway are needed to validate the protective effects of LGZGD and the protective mechanism.

## Figures and Tables

**Table 1:** Primers used in RT-qPCR.

Primers	Sequence (5′⟶3′)	
Keap1	Forward	5′-CTTCGGGGAGGAGGAGTTCT-3′
	Reverse	5′-GGGCAGTCGTATTTGACCCA-3′

HO-1	Forward	5′-TCTGCAGGGGAGAATCTTGC-3′
	Reverse	5′-TTGGTGAGGGAAATGTGCCA-3′

Nrf2	Forward	5′-TTTGTAGATGACCATGAGTCGC-3′
	Reverse	5′-TGTCCTGCTGTATGCTGCTT-3′

GAPDH	Forward	5′-AAGAGGGATGCTGCCCTTAC-3′
	Reverse	5′-ATCCGTTCACACCGACCTTC-3′

**Table 2:** Antibodies used in the study.

Antibody	Suppliers	Cat. no.	Dilution
Caspase-3	CST	9662	1 : 1000
Bcl-2	Abcam	ab196495	1 : 1000
Bax	Abcam	ab189491	1 : 1000
Keap1	Abcam	ab139729	1 : 1000
HO-1	Abcam	ab6046	1 : 2000
Nrf2	Abcam	ab137550	1 : 1000
Lamin A	Abcam	ab8980	1 : 2000
GAPDH	Abcam	ab181602	1 : 1000
Goat anti-rabbit IgG H&L(HRP)	Abcam	ab6721	1 : 10000
Rabbit anti-mouse IgG H&L(HRP)	Abcam	ab6728	1 : 10000

## Data Availability

The data used to support the findings of this study are available upon request by contact with the corresponding author.
